# Genotyping of Two Mediterranean Trout Populations in Central-Southern Italy for Conservation Purposes Using a Rainbow-Trout-Derived SNP Array

**DOI:** 10.3390/ani11061803

**Published:** 2021-06-17

**Authors:** Valentino Palombo, Elena De Zio, Giovanna Salvatore, Stefano Esposito, Nicolaia Iaffaldano, Mariasilvia D’Andrea

**Affiliations:** 1 Dipartimento Agricoltura, Ambiente e Alimenti, Università degli Studi del Molise, Via de Sanctis snc, 86100 Campobasso, Italy; abg@unimol.it (V.P.); elena.dezio@studenti.unimol.it (E.D.Z.); g.salvatore5@studenti.unimol.it (G.S.); nicolaia@unimol.it (N.I.); 2 Mediterranean Trout Research Group, Via Porali 3, 42037 Ventasso, Italy; dott.stefanoesposito@gmail.com

**Keywords:** Mediterranean trout, genetic diversity, single nucleotide polymorphism array, introgressive hybridization, biodiversity preservation

## Abstract

**Simple Summary:**

Mediterranean trout is one of the most threatened freshwater fish at risk of extinction. A complex pattern of climatic and anthropogenic pressures has dramatically compromised its biodiversity. In particular, the introduction of non-native trout represents one of the most important threats with negative impact on intraspecific diversity of native populations. The introgressive hybridization between native and alien trout reduces the fitness of native trout and creates hybrid swarms, resulting in native genome extinction. Recently, several conservation projects have been proposed to restore the genetic integrity status of native Mediterranean trout. In this study, we report the first use of the Affymetrix 57 K rainbow-trout-derived SNP array in research on Italian Mediterranean trout populations. The results provide insight into the genetic relationships and spatial distribution of two trout populations inhabiting the Volturno and Biferno rivers (Central-Southern Italy) and provide useful information for the identification of a fine-scale genetic structure, as well as the determination of subpopulations and their related habitats. These data are crucial to undertake effective conservation and management strategies with the aim to preserve native trout and recover autochthonous genetic heritage in such rivers. Overall, our outcomes support the use of the rainbow-trout-derived SNP array to identify SNPs that are informative in relation to the Mediterranean trout genome.

**Abstract:**

Mediterranean trout is a freshwater fish of particular interest with economic significance for fishery management, aquaculture and conservation biology. Unfortunately, native trout populations’ abundance is significantly threatened by anthropogenic disturbance. The introduction of commercial hatchery strains for recreation activities has compromised the genetic integrity status of native populations. This work assessed the fine-scale genetic structure of Mediterranean trout in the two main rivers of Molise region (Italy) to support conservation actions. In total, 288 specimens were caught in 28 different sites (14 per basins) and genotyped using the Affymetrix 57 K rainbow-trout-derived SNP array. Population differentiation was analyzed using pairwise weighted F_ST_ and overall F-statistic estimated by locus-by-locus analysis of molecular variance. Furthermore, an SNP data set was processed through principal coordinates analysis, discriminant analysis of principal components and admixture Bayesian clustering analysis. Firstly, our results demonstrated that rainbow trout SNP array can be successfully used for Mediterranean trout genotyping. In fact, despite an overwhelming number of loci that resulted as monomorphic in our populations, it must be emphasized that the resulted number of polymorphic loci (i.e., ~900 SNPs) has been sufficient to reveal a fine-scale genetic structure in the investigated populations, which is useful in supporting conservation and management actions. In particular, our findings allowed us to select candidate sites for the collection of adults, needed for the production of genetically pure juvenile trout, and sites to carry out the eradication of alien trout and successive re-introduction of native trout.

## 1. Introduction

The brown trout is a wide-ranging species, naturally distributed in Eurasia and North Africa, that exhibits high genetic, ecological and morphological variability [1]. The Mediterranean trout (*Salmo trutta* complex) represents one of the five main genetic lineages for European brown trout [2]. In Italy, although the taxonomy is still controversial, the Mediterranean trout is referred to as *Salmo cettii* syn. *Salmo macrostigma* [3,4,5,6,7,8] and, unfortunately, it is one of the most highly threatened freshwater fish at risk of extinction [3,7,9]. Its biodiversity is declining rapidly due to a complex pattern of climatic and anthropogenic pressures [10,11], and it is currently listed in the Italian IUCN Red List as ‘critically endangered’ (www.iucn.it, accessed on: 31 March 2021).

In particular, the introduction of non-native trout represents one of the most important threats for Mediterranean trout [5,12]. The negative effects of such practice on native specimens have been long recognized particularly in terms of competition, predation and introgressive hybridization [13,14]. Nevertheless, over the years, resource managers have adopted massive allochthonous restocking initiatives for balancing the effects of recreational overfishing. This had dramatically compromised the genetic variability of many Mediterranean trout native populations [3,12], thus several conservation projects have been recently proposed to restore native trout intraspecific biodiversity [3,5,15,16,17].

In this general context, it must be emphasized that planning an effective and evidence-based conservation project is a quite complex process [18] that notably passes through a clear characterization of genetic population structure [19,20]. This is especially important for the stream-living environment, where geological features such as waterfalls or barriers may cause further genetic differentiation between upstream and downstream populations [21], and where the scenario is further complicated by high hybridization level, as a consequence of restocking practices with hatchery-reared trout [7].

The selection of hybrids based only on morphomeristic characteristics has been long recognized as insufficient to provide a clear taxonomic information/classification, and several biochemical or genetic methods have been traditionally adopted [22,23]. With this aim, the genetic screening on fish communities has commonly relied on the use of allozymes, mitochondrial markers, PCR-based molecular markers, such as amplified fragment length polymorphism or restriction fragment length polymorphism (RFLP) [7,24,25,26,27,28]. More recently, microsatellite markers have been extensively used, revolutionizing the study of population structure for fishery conservation genetics and management purposes [29]. In light of this, it is not surprising that several research studies for the genetic characterization of Italian Mediterranean trout populations have also taken advantage from this approach with interesting results [30,31,32,33,34].

Nevertheless, due to improvements in the speed, cost and accuracy of next generation sequencing, an increasing popularity of single nucleotide polymorphism (SNP) analysis is widely recognizable in many research areas, including evolutionary, ecological and conservation studies [35,36]. In particular, the availability of several SNP-array platforms guarantees robustness and automation compared to the older microsatellite analysis [36] and allows us to genotype individual samples with a large number of SNPs in a cost-effective manner [36,37]. Various high-density SNP chips have been developed in recent years for many model species, including commercially important salmonid species, such as rainbow trout and Atlantic salmon [38,39,40,41]. Despite the limited genomic data resources available for other salmonids, the high sequence similarity between them has indicated that SNP microarrays may be suitable for the study of any member of this fish family, trout included [42,43,44].

In this work, we investigated the genetic integrity status of Mediterranean trout populations in the Biferno and Volturno rivers, two Central-Southern Italian basins with a different drainage pattern (i.e., Adriatic and Tyrrhenian). Although trout inhabiting these two rivers are of particular concern for biodiversity conservation, no relevant conservation practices have been adopted in the past nor are there any previous genetic reports available. Thus, this study aimed to give insight into genetic relationships and spatial distribution of trout population inhabiting these rivers in order to provide useful information to be considered in conservation and management activities. In this research, the use of the 57 K rainbow-trout-derived SNP array (Affymetrix) [38] for the genetic characterization of a native Italian trout population was reported for the first time.

## 2. Materials and Methods

### 2.1. Sampling

**Figure 1:**
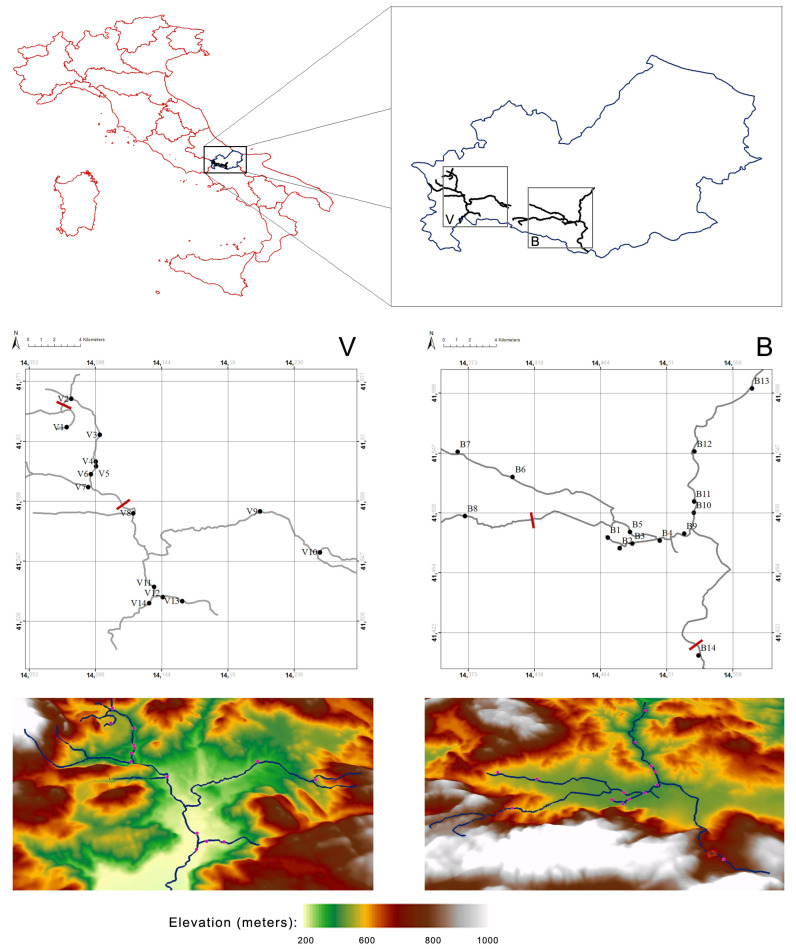
Maps showing trout sampling locations in Volturno (V) and Biferno (B) rivers. Location codes are detailed in Table S1. Red lines represent natural and/or artificial impassable barriers.

Sampling was carried out in the Biferno and Volturno rivers, the main basins of Molise (Central-Southern Italy region). For each of these watercourses, sample sites were chosen based on accessibility, survey records, habitat suitability and river connectivity within the LIFE17 NAT/IT/000547 conservation Project area. In the study area, the main threats for native trout populations are represented by habitat alterations and activities of restocking with domestic trout mainly for recreational fishing purpose. In total, 14 interesting sites were identified for each basin (Table S1; Figure 1), and 288 individuals were collected, equally subdivided between the Biferno and Volturno basins. The individuals were captured between October 2018 and November 2018, outside the reproductive season, by using a backpack electrofishing unit. A single-pass electrofishing was performed over more than 100 m length intervals at each locality to reduce the probability of repeatedly sampling family. A portion of adipose fin tissue was collected from live animals, previously anesthetized with clove powder, to avoid their sacrifice. Samples were taken using sterile technique preserved and stored in 95% ethanol (EtOH) at −20 °C until DNA isolation. Floy tags, known as spaghetti-like tags, were implanted into some adult trout to monitor movements and assess possible migratory pattern. At the end of the field activities, all specimens caught were released in the original sampling sites. All experiments were conducted with the appropriate permits of the competent authorities (Molise Region, protocol number 3969, 3 August 2018) according to the current regulations on the protection of the species, biosecurity, protocols of sampling of fresh water and animal welfare.

### 2.2. DNA Isolation, SNP-Array Genotyping and Quality Assessment

Individual genomic DNA was isolated from fin tissue fragments preserved in ethanol (~20 mg) that was carefully dissected with a sterile scalpel using DNeasy Blood and Tissue Kit^®^ (QIAGEN, Hilden, Germany) following manufacturers’ protocols and quality checked by agarose gel and BioPhotometer (Eppendorf, Hamburg, Germany).

One hundred nanograms of genomic DNA for each sample were sent to the genotyping facility (Laboratorio Genetica e Servizi Agrotis, Cremona, Italy) for marker analysis. Genotyping was performed by the 57 K rainbow trout Axiom SNP array [38]. Axiom Analysis Suite and PLINK v1.09 software [45] were used for sample and SNP quality control (QC), following the manufacturer’s best practice workflow recommendations (https://www.thermofisher.com, accessed on: 2 October 2020). A no-call threshold of 0.05 was applied and, to avoid ‘missing data’ problems, the 90% threshold for missing data was adopted. SNPs with a minor allele frequency (MAF) below the 0.01 threshold were discarded in order to prevent errors due to the presence of rare alleles. QC was performed considering all populations together and/or within each population separately to perform genetic analysis considering all or single basin samples.

### 2.3. Genetic Polymorphism

The number of polymorphic loci, the mean number of alleles, and the observed and expected heterozygosity (Ho and He) for all loci and for each population were estimated in Arlequin 3.5.2.2 software [46]. Departures from the Hardy–Weinberg equilibrium (HWE) were tested using the Markov Chain Monte Carlo random algorithm, a regime of 1,000,000 steps in Markov chain randomization and 1,000,000 dememorization steps was applied, and the significance was assessed with *p*-value < 0.05 in Arlequin.

### 2.4. Population Differentiation

Population-specific F_IS_, pairwise weighted F_ST_, and overall F-statistic [47] were estimated by locus-by-locus AMOVA (Analysis of Molecular Variance) after 10,000 permutations and significance level *p*-value < 0.05 in Arlequin. To test the role of barriers on the protection of brown trout native genetic diversity and to investigate the genetic structure within each basin, F-statistics were also applied considering each river separately. In particular, F_ST_ comparisons involving groups of samples according to the sampling locations were carried out. A sequential Bonferroni-type method was employed to correct for multiple significance tests. The two basins were also tested for patterns of isolation-by-distance (IBD), by using a Mantel test [48] with 10,000 permutations in Arlequin software, considering pairwise F_ST_ matrices and geographic distances between sampling locations.

### 2.5. Population Structure Analysis

With the aim to give insight into the genetic relationships and spatial distribution of the two trout populations, genetic distance among sampling locations was investigated by Principal Coordinates Analysis (PCoA) with GenAlex software v.6.503 [49,50] considering each basin separately. PCoA was also performed for visualizing the genetic relationship among all samples. Furthermore, the genetic ancestry was estimated using ADMIXTURE software v.1.3.0 [51] in order to identify distinct genetic populations, assuming K values from 1 to 10. The most likely number of clusters was identified based on the lowest five-fold cross validation error [51]. The results were plotted in R environment [52] and ADMIXTURE plots were generated with BITE R package [53]. To further support ADMIXTURE clustering outcomes, discriminant analysis of principal component (DAPC) [53] was conducted for each river using the adegenet R package [54]. DAPC is a multivariate method specifically designed to identify clusters of genetically related individuals by an iterative K-means approach that allows one to find the optimal number of clusters. This method does not rely on a specific population genetic model and it is free of assumptions about Hardy–Weinberg equilibrium.

### 2.6. PCR-RFLP Genotyping

All 288 samples were genotyped for 16 S rDNA, and LDH-C1* genes according to McMeel et al. [54] and Chiesa et al. [55], as a further control analysis for 100% non-native trout identification. Briefly, the nuclear gene LDH-C1* was amplified using the Ldhxon3F/Ldhxon4R primer pair and then digested with *BslI*. The resulting restriction patterns distinguish homozygote Atlantic (Atl) or Mediterranean (Med) samples from the heterozygote hybrids. A 100 bp band identifies the Med allele, while a 90 bp band recognizes the allele of Atlantic taxa [54]. The 16S rDNA was also amplified using universal primers 16Sar/16Sbr and digested with *RsaI* endonuclease, which recognizes the Atl sequence and generates two differently sized fragments, whilst the absence of the *RsaI* restriction site in the Mediterranean lineage is revealed by a single electrophoretic band.

## 3. Results

### 3.1. SNP Quality Assessment

In total, 288 DNA samples were analyzed using a 57 K SNP array [38]. No loci failed the amplification, and 35,778 SNPs were considered according to the manufacturer’s best practice workflow recommendations. After QC, in total, 920 loci were retained for further analyses encompassing all samples together, whereas 871 and 828 loci were used for further analyses within the Biferno and Volturno rivers and notably to test the differences among sampling sites.

### 3.2. Genetic Differences between Populations

**Figure 2:**
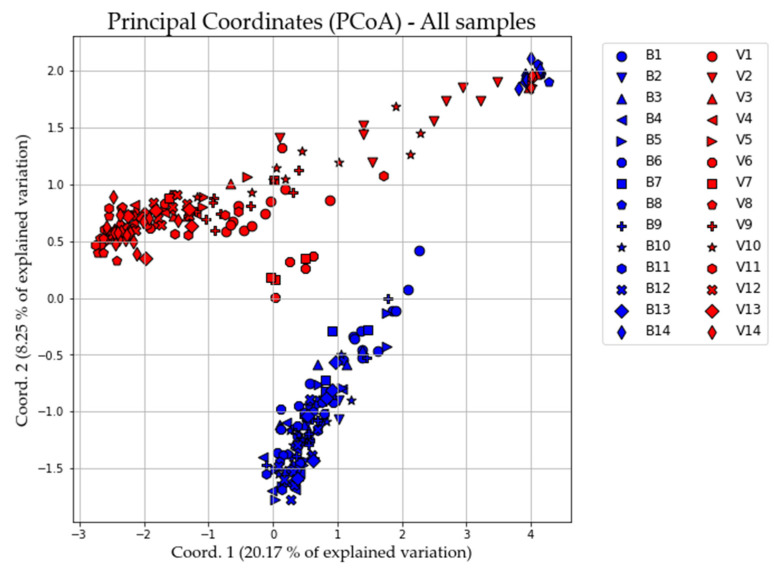
Principal coordinates analysis (PCoA) performed considering all samples. Sample locations and codes are detailed in Table S1.

Considering all samples, the overall inbreeding coefficient F_IS_ obtained by AMOVA was high (0.109) and statistically significant (*p*-value < 0.001). The average F_ST_ over all 920 loci for the two studied populations was 0.121; in turn, pairwise F_ST_ was significant and reached 0.123, indicating a high level of differentiation. The highest percentage of variation was detected within individuals 78.3% (Table S2). AMOVA was performed for all three possible scenarios: “All samples”, “Sampled in Biferno” and “Sampled in Volturno”. In all scenarios, the largest amount of variance had its source in within-individuals diversity (Table S2; *p*-value < 0.001). PCoA multivariate results showed a clear split between the analyzed populations and clustering of all samples (Figure 2).

### 3.3. Population Differentiation and Structure Analysis in the Biferno River

**Figure 3:**
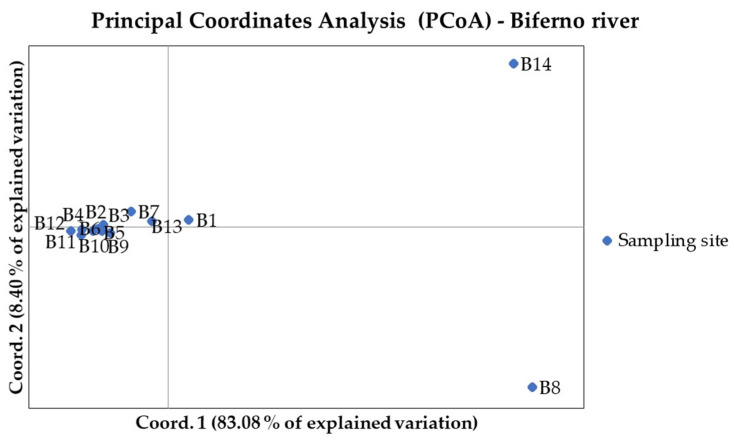
Principal coordinates analysis (PCoA) performed considering sampling sites in Biferno river. Sample locations and codes are detailed in Table S1.

**Figure 4:**
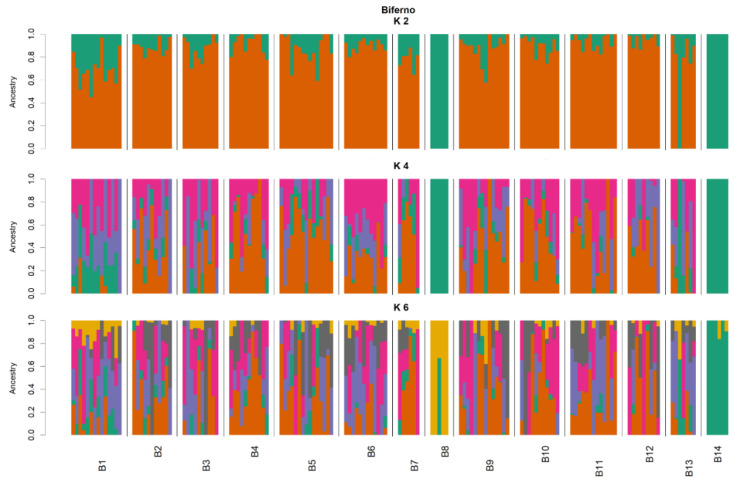
ADMIXTURE results at K = 2, K = 4 and K = 6 on Biferno genotyping data. Assignment of single individuals (thin vertical bars) to different clusters. Different colors identify different clusters. The reconstruction at K = 2 had the smallest cross-validation error (see Figure S1).

**Figure 5:**
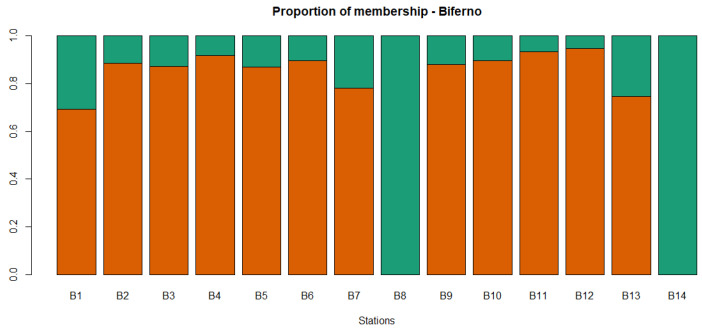
Proportion of membership of each sampling locations in each of the 2 clusters estimated in ADMIXTURE for the Biferno river. Orange and green colors represent group 1 and group 2, respectively.

For the 920 loci, the mean Ho for the population from the Biferno river was 0.219 (±0.182) and values ranged from 0.00 to 1.00, whereas the He amounted to 0.235 (±0.185) and ranged from 0.007 to 0.502 (Table 1). In total, 95 loci resulted monomorphic in Biferno, whereas departure from HWE was observed at 205 (22.28% of total). Focusing on within river analyses, for 871 loci within the Biferno river population (Table 2), the mean Ho ranged from 0.278 of B11 station to 0.424 of B8 station, whereas in the same stations, the He was 0.276 and 0.400, respectively. The highest number of monomorphic loci was detected in B8 station (599), whereas the lowest in B9 station (219). The highest number of loci deviating from HWE was observed in B9 station (33 loci), whereas the lowest in B8 station (4 loci). The average F_ST_ over all 871 loci for Biferno river population was 0.08. The highest significant pairwise F_ST_ (0.430) was detected for the B8 vs. B12 comparison, whereas the lowest (0.025) in B1 vs. B9 comparison (Table 3). Pairwise F_ST_ and pairwise geographic distances were non-significantly correlated in the Biferno river (r_Mantel_ = 0.40, *p*-value = 0.06). PCoA multivariate results helped to identify sampling sites with distinct genetics (Figure 3). In this regard, PCR-RFLP genotyping results (Table S3), which provided a control for the 100% Atl lineage, revealed that samples in B8 and B14 sites were entirely characterized by non-native genetic make-up (high frequency of LDH-C1*90 and of Atlantic 16S rDNA alleles; Table S3). Multivariate analyses were further confirmed by the assignment values obtained from the ADMIXTURE tests [51] that showed the main decrease in the five-fold cross validation error at the optimal genetic clusters of K = 2 for the Biferno river (Figure S1) and clearly separated Mediterranean trout from Atlantic lineage (Figure 4 and Figure 5). This scenario was also evident in the DAPC outcomes (Figure S2). In particular, these results confirmed the complete overlapping between all Biferno sampling sites, except for B8 and B14 locations. Furthermore, DAPC results helped to assess the number of clusters identified with ADMIXTURE analysis. In this case, the choice of the ‘optimal’ number of clusters was made based on BIC improvement (Figure S2) and it was congruent with the scenario depicted by ADMIXTURE analysis.

### 3.4. Population Differentiation and Structure Analysis in the Volturno River

**Figure 6:**
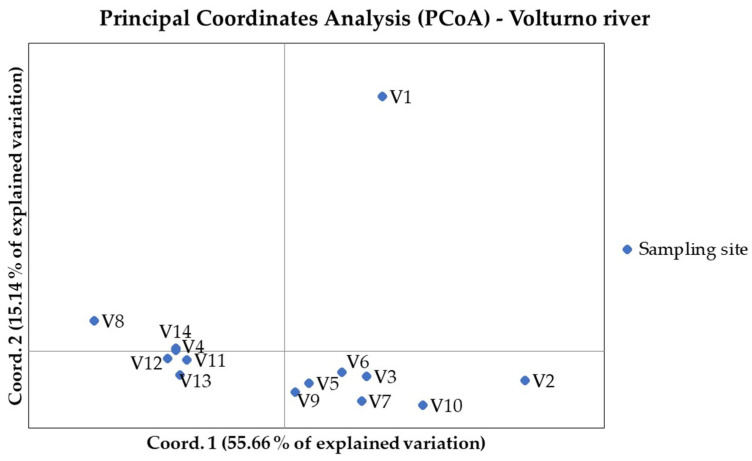
Principal coordinates analysis (PCoA) performed considering sampling sites in Volturno river. Sample locations and codes are detailed in Table S1.

**Figure 7:**
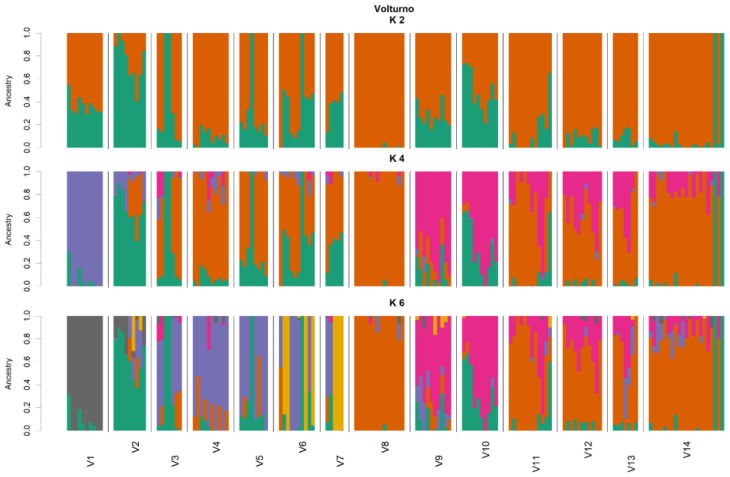
ADMIXTURE results at K = 2, K = 4 and K = 6 on Volturno genotyping data. Assignment of single individuals (thin vertical bars) to different clusters. Different colors identify different clusters. The reconstruction at K = 4 had the smallest cross-validation error (see Figure S1).

**Figure 8:**
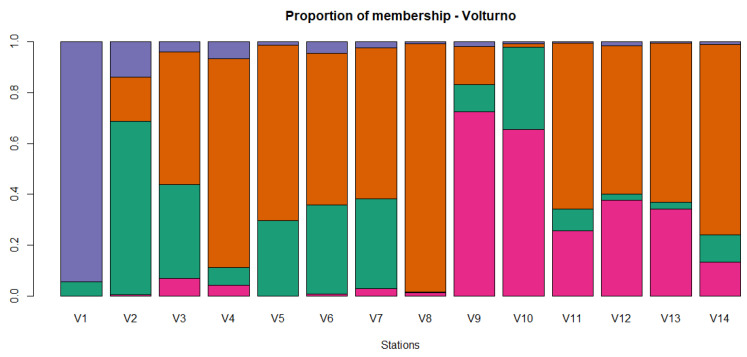
Proportion of membership of each sampling locations in each of the 4 clusters estimated in ADMIXTURE for the Volturno river. Orange, green, pink and violet colors represent group 1, 2, 3 and 4, respectively.

In the Volturno river, for the 920 loci, the mean Ho and He were slightly lower than Biferno river, 0.169 (±0.156) and 0.200 (±0.165), and ranged from 0.00 to 1.00 and from 0.007 to 0.502. In total, 320 monomorphic loci (34.78% of total) resulted, 131 of which in common with the Biferno river (Table 1). Focusing on within river analyses, for 828 loci in the Volturno population (Table 4), the mean Ho ranged from 0.187 of V14 station to 0.351 of V1 station; the highest He (0.371) was detected in V3 station, whereas the lowest (0.229) in V14 station. The highest number of monomorphic loci was detected in V8 station (461) and the lowest in V6 station (200). In turn, the highest number of loci deviating from HWE was observed in V14 station (120 loci), whereas the lowest in V5 station (11 loci). The average F_ST_ over all 828 loci for the Volturno river population was 0.120; in turn, the highest and lowest significant pairwise F_ST_ reached 0.437 in V2 vs. V8 comparison and 0.048 in V4 vs. V12 comparison, respectively (Table 5). The IBD pattern was significant for the Volturno river (r_Mantel_ = 0.31, *p*-value = 0.01), suggesting the presence of genetic discontinuity. PCoA multivariate results helped to identify sampling sites with distinct genetics (Figure 6). Multivariate analysis was further confirmed by the assignment values obtained from the ADMIXTURE tests [51] that showed the main decrease in the five-fold cross validation error at the optimal genetic clusters of K = 4 for the Volturno river (Figure S1; Figure 7 and Figure 8). This scenario was also evident in the DAPC outcomes (Figure S2), which also helped to assess the number of clusters identified with ADMIXTURE analysis. Compared to the Biferno river, PCoA and DAPC results revealed a more complex pattern in the Volturno, mainly characterized by the presence of only few sites partially overlapped and by the evident isolation of V1 sampling location, in which PCR-RFLP genotyping results suggested the high occurrence of native genetic make-up (Table S3). Taken together, these observations supported the hypothesis of a higher intra-river genetic discontinuity in the Volturno river.

## 4. Discussion

The introduction of non-native cultured fish has been recognized as one of the most important threats to native trout populations in many countries [12], including Italy [5]. Recently, several conservation projects have been proposed to protect Mediterranean lineage trout populations [3,5,15]. In this regard, it must be emphasized that the importance of such projects goes beyond the single species conservation purpose considering also adverse ecological impacts of the stocked trout on ecosystem communities [56]. In this study, a 57 K SNP array developed for rainbow trout was used for the first time to analyze the genetic relationship between Mediterranean trout specimens, from two rivers in the Molise region (in Central-Southern Italy). The situation under study is characterized by the human influence on wild Mediterranean trout population, since restocking practices with Atlantic lineage trout have been conducted over the years mainly to enhance recreational fisheries. The expected scenario was to observe an extensive introgressive hybridization of hatchery genetic markers into native populations. The main aim of this research was to collect information on the genetic integrity status of native trout species in order to provide support for conservation activities within the LIFE17 NAT/IT/000547 Project, since no previous data were available.

Along with the strictly related population genetics outcomes, from a general point of view, our results firstly demonstrated that an SNP array derived from rainbow trout genome can be successfully used for Mediterranean trout genotyping. Indeed, ~63% of total loci were successfully amplified and passed quality control when DNA of Mediterranean trout was genotyped. Furthermore, despite a rather small number of polymorphic loci being identified in our populations (i.e., 920 SNPs at MAF > 0.01), it must be recognized that such number has been sufficient to reveal a fine-scale genetic structure and to separate the trout populations under study as shown by ADMIXTURE, PCoA and DAPC analyses (Figure 3, Figure 4, Figure 6, Figure 7 and Figure S2). Obtained data are even more than satisfactory when taking previous microsatellite results into consideration, since they seemed to be not fully effective to differentiate the Mediterranean trout from hatchery stocks [30,57].

Overall, genetic analyses presented here showed a clear genetic differentiation between Biferno and Volturno samples; indeed, the F_ST_ value was significant and reached 0.123 across all 920 polymorphic SNPs. This divergence was also well-represented in PCoA results by a marked split between the two analyzed populations (Figure 2). Focusing on this result, which showed the clustering of all samples from the two rivers, an overlapping region was recognizable, representing the Atl specimens detected in both rivers as revealed by PCR-RFLP and ADMIXTURE analyses (Table S3, Figure 4 and Figure 7). Our results confirmed that Atl genome are widespread in wild trout populations of the study area; this was not surprising considering that several testimonies have reported domestic restocking activities in both rivers. Furthermore, the rate of 16S Atl locus ranged from 0 to 100%, and a similar pattern was detected for LDH-C1*90 allele (Table S3). The ADMIXTURE outcomes (Figure 4, Figure 5, Figure 7 and Figure 8) supported this observation, providing a pattern of genetic differentiation congruent with PCoA and DAPC outcomes (Figure 3, Figure 6 and Figure S2) and notably confirming the presence of two Biferno stations (B8 and B14) entirely characterized by allochthonous specimens. These sites were located in the Biferno tributaries, isolated from the main river rod by the presence of natural and artificial impassable barriers (Figure 1). The spatial isolation of such locations, recognizable also by significant and high pairwise F_ST_ values (Table 3), was noteworthy and suggested that the eradication of the alien population and the simultaneous re-introduction of pure native trout might be an effective conservation strategy for this site (i.e., tributaries). In general, our genetic data suggested a high gene flow between Biferno main rod sampling sites that can be grouped together genetically. In fact, the K = 2 was the optimal number of clusters in the Biferno population (Figures S1 and S2), which was consistent with the expected scenario of hybridization between native and invasive trout. Our results provided evidence that Biferno population was genetically homogenous; indeed, excluding B8 and B14, the membership coefficient for native genetics ranked from 44.9% to 100%, with an average across samples of 89.0%. PCoA results confirmed this scenario, showing sampling sites close together (Figure 3). All this suggested a high migration rates within the Biferno river, and this was consistent with observations obtained by spaghetti-like tags tracking system, which indicated a quite range of mobility of adults monitored in Biferno (>10 Km). This was not surprising considering the absence of natural and artificial impassable barriers along the main rod encompassed by the project area. The scenario depicted above was also corroborated by IBD outcomes that indicated non-significantly correlation between F_ST_ and geographic distance matrices for the Biferno river. It is also important to highlight that a relatively resident lifestyle was observed at B1, which appeared to be the most introgressed area in the Biferno river, and B7 spawning sites. This observation was in line with the comparatively higher percentage of Atl genome detected in such locations compared to the average across all sites, excluding B8 and B14 specimens (Figure 4 and Figure 5). The intrinsic characteristics of these locations (i.e., small upstream spawning sites) suggested that the eradication of alien trout species and the simultaneous re-introduction of pure native trout could be an appropriate conservation strategy for such sites.

As already stated, our results provided evidence that the allochthonous genome is widely spreading into the wild of both rivers; however, a different pattern of introgression seemed to be recognizable within the two basins. Indeed, whereas the analysis with ADMIXTURE for Biferno population did not indicate a clear-cut distinction between native and alien clusters (except for B8 and B14 locations), a more complex pattern was detected in Volturno, where K = 4 was the optimal genetic cluster repartition (Figures S1 and S2). The inspection of individual admixture proportion in the Volturno river evidenced the presence of two stations (i.e., V1 and V8) with a high percentage of individuals classifiable as pure native. This result was also supported by PCR-RFLP outcomes (Table S3) and ADMIXTURE analysis suggested the hypothesis of the existence of at least two major genetically differentiated groups (metapopulation) of native trout in the Volturno river. V1 and V8 sampling sites were characterized by different genetics (Figure 7 and Figure 8). Excluding the 100% allochthonous specimens, the member coefficient of native genetics in Volturno ranged from 7.9% to 100%, with an average percentage across the samples of 79.2%, indicating a more heterogeneous scenario compared to Biferno. In this regard, it is noteworthy to highlight that landscape features probably have had a strong effect in shaping such peculiar metapopulation structure. In fact, V1 station is the natural source of the Volturno river isolated by a vertical waterfall (~10 m) from all downstream sample sites. As revealed by the ADMIXTURE results (Figure 7 and Figure 8), downstream population was characterized not only by native genetics of migrants from the river source (i.e., one-way downstream gene flow) but also by alien (i.e., Atlantic) genetic make-up derived from headwater streams. This aspect was particularly evident in the V2 sampling site (Figure 8, Table S3) and suggested the eradication of alien trout as a possible strategy for V2 location, since this area appeared to be the most introgressed in the Volturno river. Moreover, it is interesting to note that locations below the waterfall were characterized by a gradually greater component of the second native genetics identified in Volturno. This complex pattern seemed to suggest that any introgression between hatchery and native fish did not have a lasting impact on native fish genetic diversity in the Volturno river and supported the idea that possible temporal and behavioral differences among Mediterranean and Atlantic trout may exist, as well described by Splendiani et al. [34]. This result is very interesting considering the history of restocking with hatchery trout also in the Volturno river, which would be expected to result in a major impairment of any trace of pure native individuals. It is important to highlight that the V8 station was almost entirely characterized by the second native genetics (Figure 7 and Figure 8). This site can be considered as the ‘real’ source of anthropic origin in the Volturno river (Figure 1). Actually, much of the water captured at the natural source (V1 sampling site) for hydroelectric purpose is released at the V8 location where finally the Volturno river reaches its entire flow. The genetics observed at the V8 site can be considered as the main native genetics characterizing the entire main course of the river. This point deserves particular mention, since, as observed in the Biferno river, the native genetics were predominantly associated with the main rod of the river, whereas a more compromised situation was detectable in tributaries. This aspect appeared particularly noteworthy with regard to the different patterns usually described in similar studies where tributaries seemed to be resistant to trout hybridization and contained predominantly pure native trout, while the main river courses resulted as compromised [58,59,60]. This peculiar scenario suggested, in our case, that sites along the main course might represent promising and potential locations for the collections of adults needed for the production of genetically pure juvenile trout.

This situation was further complicated in the Volturno river by the presence of a third autochthonous genetics, mainly associated with the V9 and V10 stations (Figure 7 and Figure 8). In light of the resident and migratory life history theories [61] and considering the absence of impassable barriers with the main river course, this observation suggested the existence of potential third native genetics, typical of Volturno tributaries, characterized by resident trout. This hypothesis was also supported by IBD results, which indicated significant and moderate correlation between F_ST_ and geographic distance matrices for the Volturno river. Although peculiar processes that maintain genetic differentiation in the Volturno when physical barriers are lacking would merit further attention, no eradication strategy was proposed for Volturno tributaries’ locations in order to preserve this peculiar genetic structure.

In summary, the genetic characterization of the trout population in the Biferno and Volturno rivers allowed us to appreciate subpopulation structure and helped to define sites for the collections of pure adult spawners and sites to carry out the eradication of alien trout and successive re-introduction of Mediterranean trout. The complex pattern of naturally isolated and connected subpopulations of Mediterranean trout described in this study demonstrated the importance of considering the fine-scale genetic structure in a conservation management project.

## 5. Conclusions

This study reported the first use of SNP-array technology in research on Mediterranean trout populations in Italy. In particular, this paper aimed to explore the trout genetic structure of Biferno and Volturno rivers (in Central-Southern Italy), in order to assist trout conservation and management activities. The expected scenario in such rivers was that anthropogenic translocation of non-native trout has altered the native trout biodiversity.

Firstly, our results suggested that rainbow trout 57 K SNP array can be effective in the identification of informative SNPs in the Mediterranean trout genome, which is useful for fine-scale population genetic research. Specifically, our genetic structure outcomes allowed us to appreciate a complex metapopulation pattern notably in the Volturno river, whereas a more homogenous scenario was observed in Biferno, suggesting a high migration rate and gene flow among its subpopulations.

The fine-scale genetic characterization of these populations allowed us to select sites for the collection of adults and spawners, as well as sites to carry out the eradication of alien trout. Overall, our data provided supporting information for the improvement of genetic biodiversity restitution in Mediterranean trout populations of the Biferno and Volturno basins.

## Figures and Tables

**Table 1:** Genetic diversity for two trout population from Molise rivers. N—number of individuals, NPL—number of polymorphic loci, MNA—mean number of alleles, Ho observed heterozygosity, He—expected heterozygosity, DHWE—loci with deviation from H–W equilibrium and population specific F_IS_.

River	N	NPL	MNA	Ho	He	DHWE	F_IS_
Biferno	144	825	143.01	0.219	0.235	205	0.081
Volturno	144	850	143.26	0.169	0.200	320	0.133

**Table 2:** Genetic diversity for trout population from Biferno stations. N—number of individuals, NPL—number of polymorphic loci, MNA—mean number of alleles, Ho—observed heterozygosity, He—expected heterozygosity, DHWE—loci with deviation from H–W equilibrium and sampling site specific F_IS_. Sample locations and codes are detailed in Table S1.

Locations	N	NPL	MNA	Ho	He	DHWE	F_IS_
B1	14	641	13.94	0.315	0.314	31	−0.001
B2	11	612	10.94	0.313	0.305	28	−0.015
B3	10	607	9.95	0.310	0.311	17	0.003
B4	11	593	10.94	0.304	0.298	27	−0.010
B5	15	649	14.88	0.293	0.293	17	0.004
B6	12	592	11.92	0.321	0.306	21	−0.032
B7	6	522	5.97	0.386	0.372	6	−0.043
B8	5	272	4.93	0.424	0.400	4	−0.066
B9	14	652	13.93	0.281	0.283	33	0.014
B10	11	620	10.91	0.297	0.296	25	−0.005
B11	13	645	12.92	0.278	0.276	32	−0.005
B12	9	589	8.93	0.299	0.300	14	0.009
B13	7	557	6.97	0.300	0.353	17	0.115
B14	6	354	5.90	0.386	0.360	9	−0.064

**Table 3:** Population pairwise F_ST_ calculated considering sampling stations in the Biferno river, calculated in Arlequin. On diagonal: average number of pairwise difference within population. Above the diagonal: the *p*-value significance after Bonferroni correction. Sample locations and codes are detailed in Table S1.

	B1	B2	B3	B4	B5	B6	B7	B8	B9	B10	B11	B12	B13	B14
B1	199.249	***	ns	***	***	***	ns	***	***	***	***	***	ns	***
B2	0.024	184.515	ns	ns	ns	ns	ns	***	ns	ns	ns	ns	ns	***
B3	0.019	−0.003	186.811	ns	ns	ns	ns	ns	ns	ns	ns	ns	ns	***
B4	0.033	0.001	0.008	175.048	ns	ns	ns	***	ns	ns	ns	ns	ns	***
B5	0.029	0.000	0.015	0.007	186.605	ns	ns	***	ns	ns	ns	ns	ns	***
B6	0.031	0.005	0.009	0.005	0.008	178.478	ns	***	ns	ns	ns	ns	ns	***
B7	0.018	0.015	0.016	0.015	0.023	0.010	192.061	ns	ns	ns	***	ns	ns	ns
B8	0.262	0.381	0.378	0.408	0.363	0.392	0.347	105.778	***	ns	***	***	ns	ns
B9	0.025	−0.006	0.006	−0.007	−0.001	0.003	0.015	0.371	182.492	ns	ns	ns	ns	ns
B10	0.030	0.000	0.002	−0.005	0.001	0.008	0.018	0.386	−0.007	180.455	ns	ns	ns	***
B11	0.045	0.003	0.011	0.001	0.001	0.004	0.027	0.409	−0.001	−0.002	175.945	ns	ns	***
B12	0.046	0.004	0.010	0.010	0.006	0.010	0.031	0.430	0.009	0.005	0.008	174.046	ns	***
B13	0.001	0.015	0.015	0.022	0.005	0.016	0.012	0.308	0.008	0.011	0.022	0.023	194.560	ns
B14	0.238	0.353	0.350	0.380	0.338	0.367	0.313	0.116	0.345	0.358	0.383	0.402	0.281	123.333

*** and ns represent *p*-value < 0.001 and > 0.05, respectively.

**Table 4:** Genetic diversity for trout population from Volturno stations. N—number of individuals, NPL—number of polymorphic loci, MNA—mean number of alleles, Ho—observed heterozygosity, He—expected heterozygosity, DHWE—loci with deviation from H–W equilibrium and sampling site specific F_IS_. Samples locations and codes are detailed in Table S1.

Locations	N	NPL	MNA	Ho	He	DHWE	F_IS_
V1	10	443	9.95	0.351	0.329	25	−0.054
V2	9	611	8.91	0.345	0.327	18	−0.039
V3	7	526	6.98	0.264	0.371	42	0.230
V4	10	454	9.97	0.281	0.266	19	−0.031
V5	8	534	7.94	0.309	0.334	11	0.077
V6	10	628	9.94	0.250	0.302	36	0.136
V7	5	486	4.97	0.347	0.367	13	0.035
V8	14	367	13.94	0.269	0.244	29	−0.045
V9	10	549	9.96	0.321	0.313	23	−0.018
V10	10	545	9.95	0.341	0.350	21	0.022
V11	12	536	11.94	0.248	0.259	42	0.057
V12	11	484	10.95	0.271	0.259	20	−0.025
V13	7	413	6.97	0.320	0.295	13	−0.067
V14	21	615	20.91	0.187	0.229	120	0.189

**Table 5:** Population pairwise F_ST_ calculated considering sampling stations in the Volturno river, calculated in Arlequin. On diagonal: average number of pairwise difference within population. Above the diagonal: the p-value significance after Bonferroni correction. Sample locations and codes are detailed in Table S1.

	V1	V2	V3	V4	V5	V6	V7	V8	V9	V10	V11	V12	V13	V14
V1	144.153	***	***	***	***	***	***	***	***	***	***	***	ns	***
V2	0.216	195.588	ns	***	ns	ns	***	***	***	ns	***	***	ns	***
V3	0.121	0.093	194.198	ns	ns	ns	ns	***	ns	ns	ns	ns	ns	ns
V4	0.214	0.323	0.090	120.132	ns	ns	***	***	***	***	ns	***	***	ns
V5	0.147	0.159	−0.004	0.046	175.667	ns	ns	***	ns	ns	ns	***	***	ns
V6	0.123	0.136	0.004	0.082	0.010	187.684	ns	***	ns	ns	ns	ns	ns	ns
V7	0.170	0.169	0.040	0.138	0.047	0.002	176.067	***	ns	***	ns	ns	ns	ns
V8	0.298	0.437	0.198	0.059	0.153	0.175	0.242	88.601	***	***	***	***	***	ns
V9	0.162	0.190	0.033	0.075	0.031	0.035	0.056	0.158	170.526	***	ns	***	ns	ns
V10	0.170	0.089	0.031	0.188	0.073	0.057	0.090	0.293	0.069	189.274	***	***	***	ns
V11	0.193	0.288	0.072	0.028	0.053	0.065	0.107	0.053	0.042	0.149	137.395	ns	ns	ns
V12	0.217	0.321	0.105	0.048	0.074	0.092	0.145	0.063	0.064	0.171	0.011	124.030	ns	ns
V13	0.224	0.311	0.099	0.049	0.071	0.085	0.131	0.085	0.069	0.170	0.025	0.015	120.791	ns
V14	0.189	0.301	0.080	0.021	0.050	0.070	0.110	0.042	0.052	0.161	−0.006	0.005	0.017	139.397

*** and ns represent *p*-value < 0.001 and > 0.05, respectively.

## Data Availability

The data presented in this study are available on request from the corresponding author. Supporting data can be made available to bona fide researchers subject to a non-disclosure agreement.

## Supplementary Materials

The following are available online at https://www.mdpi.com/article/10.3390/ani11061803/s1, Figure S1: Plot of ADMIXTURE cross-validation error from K = 1 through K = 10. We chose K = 2 and K = 4 to analyze the SNP data in Biferno and Volturno rivers, respectively, as values that minimize the error. Figure S2: Discriminant analysis of principal component (DAPC) scatter plots of individuals using 871 and 828 SNP set for Biferno and Volturno rivers, respectively. In total, 20 PCs and 5 discriminant functions were retained during analyses to describe the relationship between the clusters. The bottom graphs illustrate the Bayesian Information Criterion (BIC) values for increasing values of cluster in the DAPC. Table S1: Numbers (N) of brown trout specimens examined, river and sampling location coordinates. Population density, elevation and distance from source are also provided. Table S2: Analysis of Molecular Variance (AMOVA) applying the F_ST_ estimator of Weir and Cockerham variance component, calculated for 3 scenarios: all samples, samples only in the Biferno, and only in the Volturno river. Table S3: Frequency table with genotyping results at LDH and 16S loci for each sampling locations. Samples sites and codes are detailed in Table S1.
