# Supplementary material for: Genotyping of Two Mediterranean Trout Populations in Central-Southern Italy for Conservation Purposes Using a Rainbow-Trout-Derived SNP Array
Source: Animals (Basel). 2021 Jun 17;11(6):1803. doi: 10.3390/ani11061803 (PMC8233821; doi:10.3390/ani11061803)
Supplement: Supplementary file 1 [file animals-11-01803-s001.zip › animals-1252848-supplementary.pdf]

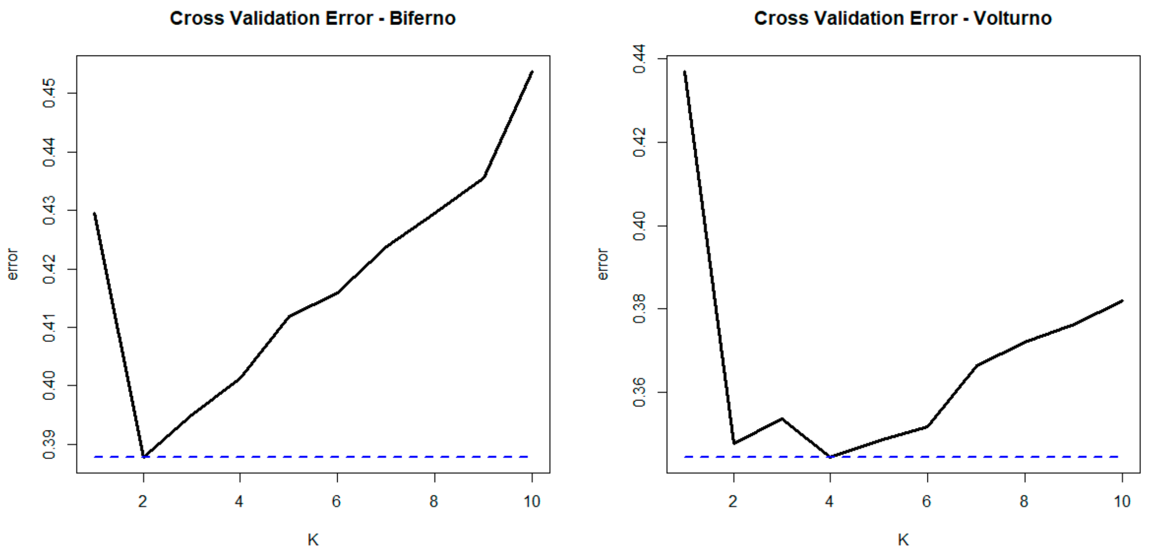

Figure S1. Plot of ADMIXTURE cross validation error from K = 1 through K = 10. We chose K = 2 and K = 4 to analyze the SNP data in Biferno and Volturno rivers respectively, as values that minimize the error.

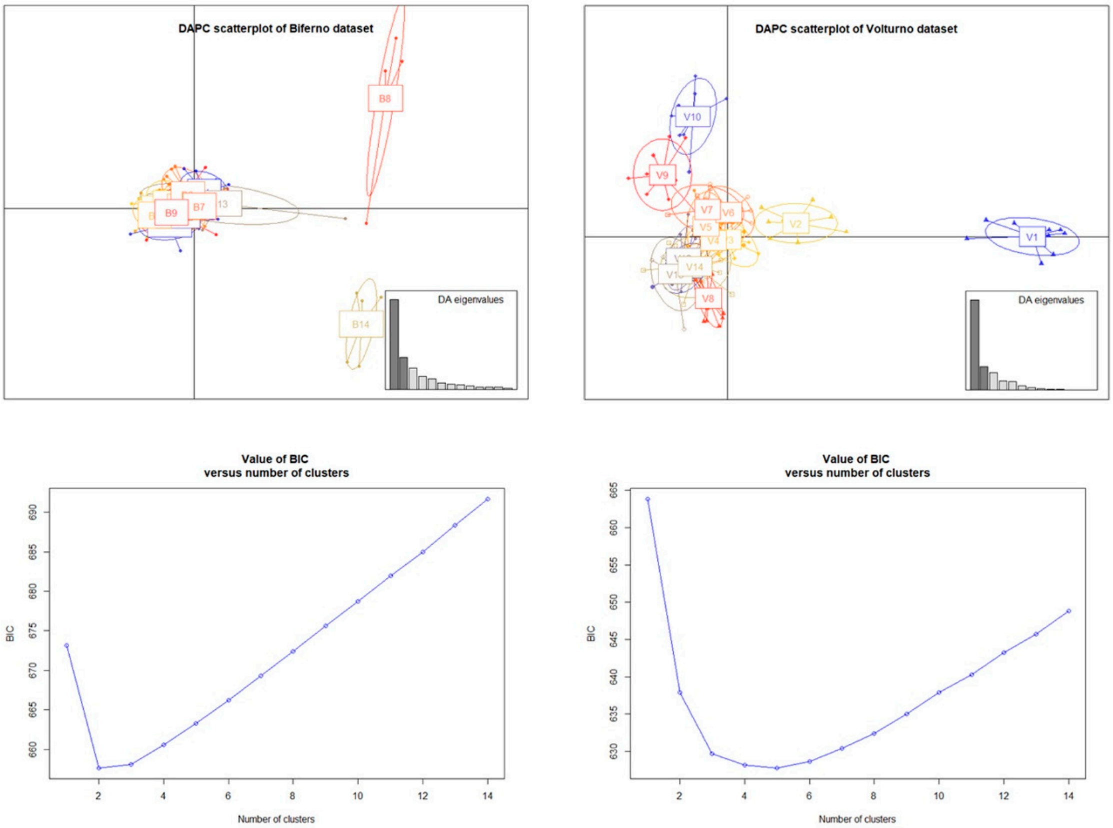

Figure S2. Discriminant analysis of principal component (DAPC) scatter plots of individuals using 871 and 828 SNP set for Biferno and Volturno rivers, respectively. 20 PCs and 5 discriminant functions were retained during analyses to describe the relationship between the clusters. The bottom graphs illustrate the Bayesian Information Criterion (BIC) values for increasing values of cluster in the DAPC.

Table S1. Numbers (N) of brown trout specimens examined, river and sampling location coordinates. Population density, elevation and distance from source are also provided.

River	Location code	Lat.	Long.	N	Population density N/m²	Elevation (m slm)	Distance from source (m)
Biferno	B1	41°28'59.51''N	14°27'58.24''E	14	0.66	486	0
	B2	41°28'48.62''N	14°28'41.63''E	11	0.25	481	0
	B3	41°28'58.48''N	14°28'59.44''E	10	0.41	478	700
	B4	41°29'11.25''N	14°30'21.97''E	11	0.17	474	3,300
	B5	41°29'37.24''N	14°29'2.17''E	15	0.16	479	13,800
	B6	41°31'49.62''N	14°24'12.22''E	12	0.27	525	3,930
	B7	41°32'52.65''N	14°21'55.55''E	6	0.23	575	0
	B8	41°30'12.54''N	14°22'13.18''E	5	0.03	636	-
	B9	41°29'28.95''N	14°31'18.80''E	14	0.17	468	6,600
	B10	41°30'26.22''N	14°31'51.76''E	11	0.07	464	8,000
	B11	41°30'49.43''N	14°31'42.93''E	13	0.17	460	9,100
	B12	41°32'53.53''N	14°31'44.06''E	9	0.06	430	13,700
	B13	41°35'29.55''N	14°34'7.38''E	7	0.02	388	20,500
	B14	41°24'33.39''N	14°31'56.54''E	6	0.01	861	-
Volturno	V1	41°38'21.4"N	14°04'43.0"E	10	0.21	552	500
	V2	41°39'23.9"N	14°04'43.9"E	9	0.04	515	3,400
	V3	41°38'04.2"N	14°06'09.3"E	7	0.01	383	7,000
	V4	41°37'00.1"N	14°05'51.7"E	10	0.01	345	9,300
	V5	41°36'50.2"N	14°05'53.3"E	8	0.01	341	-
	V6	41°36'23.3"N	14°05'48.9"E	10	0.02	329	10,500
	V7	41°35'50.6"N	14°05'49.0"E	5	0.10	322	-
	V8	41°34'46.0"N	14°07'35.0"E	14	0.01	270	15,000
	V9	41°34'40.3"N	14°12'42.3"E	10	0.01	322	-
	V10	41°33'10.9"N	14°15'11.7"E	10	-	478	-
	V11	41°31'33.4"N	14°08'29.8"E	12	0.04	233	-
	V12	41°31'20.5"N	14°08'42.4"E	11	0.28	222	-
	V13	41°31'00.7"N	14°09'24.3"E	7	0.15	249	-
	V14	41°31'8.43"N	14'8'16.41"E	21	0.03	218	23,000

Table S2. Analysis of Molecular Variance (AMOVA) applying the F_{ST} estimator of Weir and Cockerham variance component, calculated for 3 scenarios: all samples, samples only in Biferno, and only Volturno river. The results were significant at *p*-value < 0.001.

	**Among Populations**		**Among individuals within populations**		**Within individuals**	
	**Variance component**	**% variation**	**Variance component**	**% variation**	**Variance component**	**% variation**
All samples	12.58	12.15	9.90	9.55	81.10	78.30
Samples in Biferno	7.93	8.04	-0.37	-0.38	91.00	92.34
Samples in Volturno	10.35	12.04	3.41	3.96	72.22	84.00

Table S3. Frequency table with genotyping results at LDH and 16S loci for each sampling locations. Samples sites and codes are detailed in Table S1.

River	Locations	LDH			16S	
		100/100	100/90	90/90	Med	Atl
Biferno	B1	29%	64%	7%	64%	36%
	B2	27%	73%	0%	73%	27%
	B3	50%	40%	10%	90%	10%
	B4	27%	45%	27%	91%	9%
	B5	60%	40%	0%	80%	20%
	B6	75%	25%	0%	83%	17%
	B7	33%	50%	17%	100%	0%
	B8	0%	0%	100%	0%	100%
	B9	21%	64%	14%	79%	21%
	B10	45%	45%	9%	91%	9%
	B11	54%	46%	0%	77%	23%
	B12	89%	11%	0%	89%	11%
	B13	29%	57%	14%	86%	14%
	B14	0%	0%	100%	0%	100%
Volturno	V1	50%	50%	0%	100%	0%
	V2	11%	33%	56%	33%	67%
	V3	57%	14%	29%	71%	29%
	V4	90%	10%	0%	90%	10%
	V5	50%	38%	13%	88%	13%
	V6	80%	10%	10%	80%	20%
	V7	60%	40%	0%	60%	40%
	V8	43%	57%	0%	100%	0%
	V9	50%	40%	10%	80%	20%
	V10	40%	40%	20%	50%	50%
	V11	67%	25%	8%	92%	8%
	V12	91%	9%	0%	82%	18%
	V13	71%	29%	0%	43%	57%
	V14	86%	5%	10%	90%	10%
